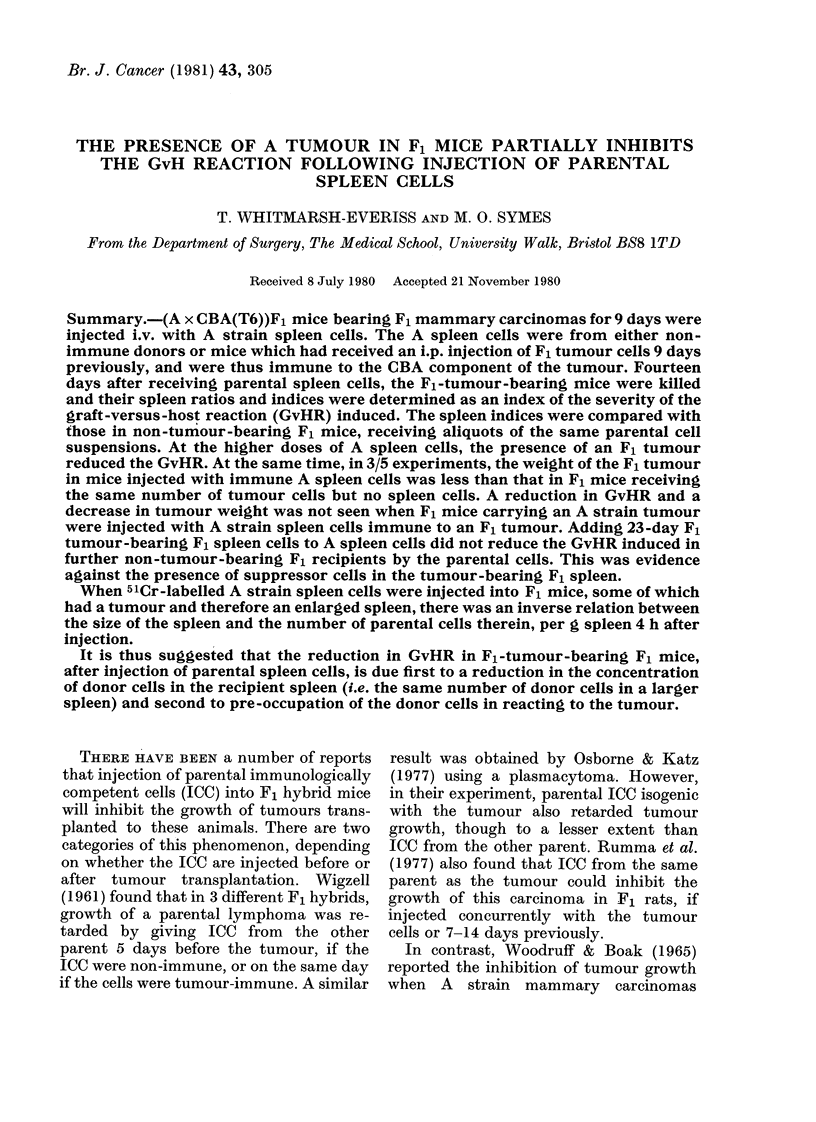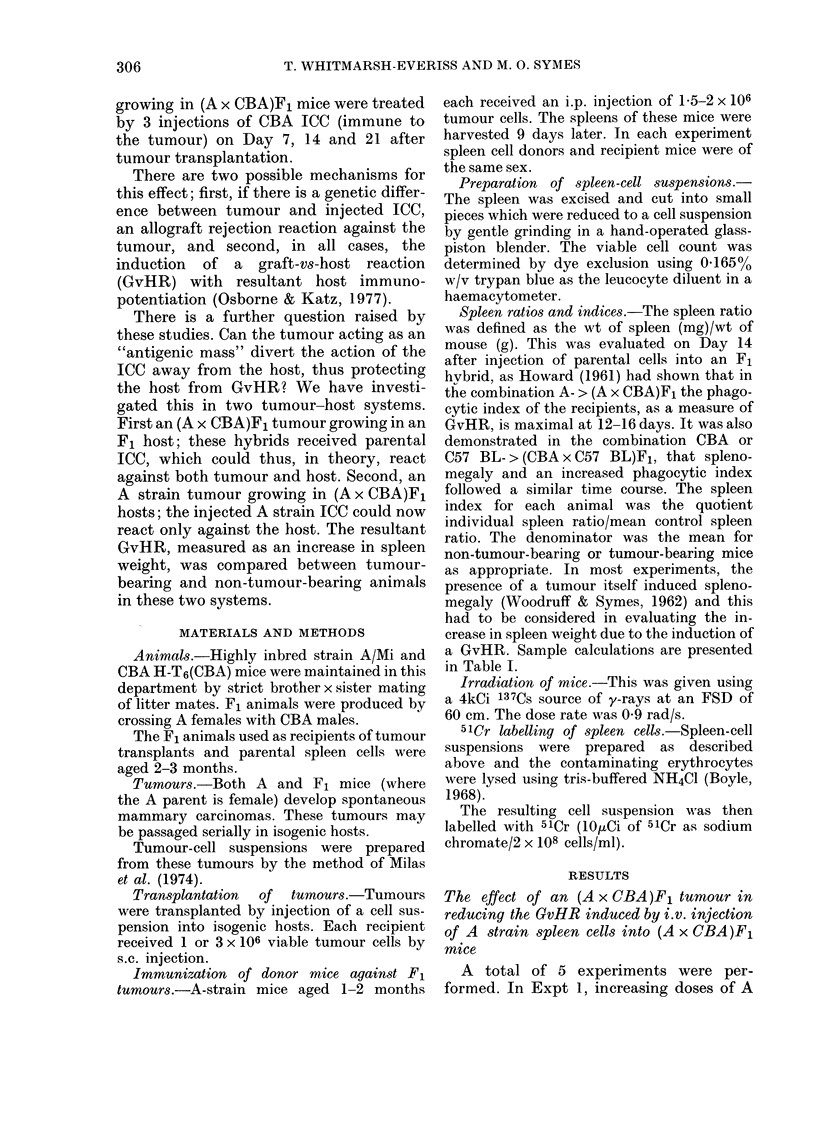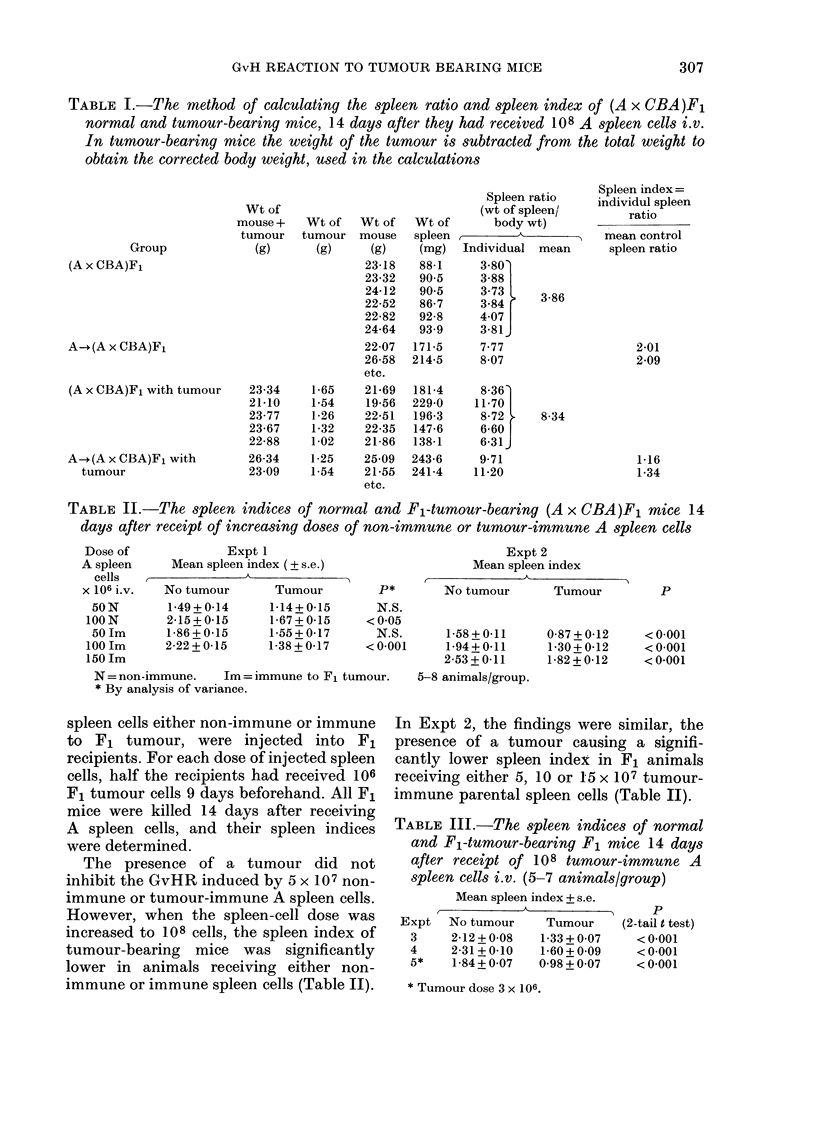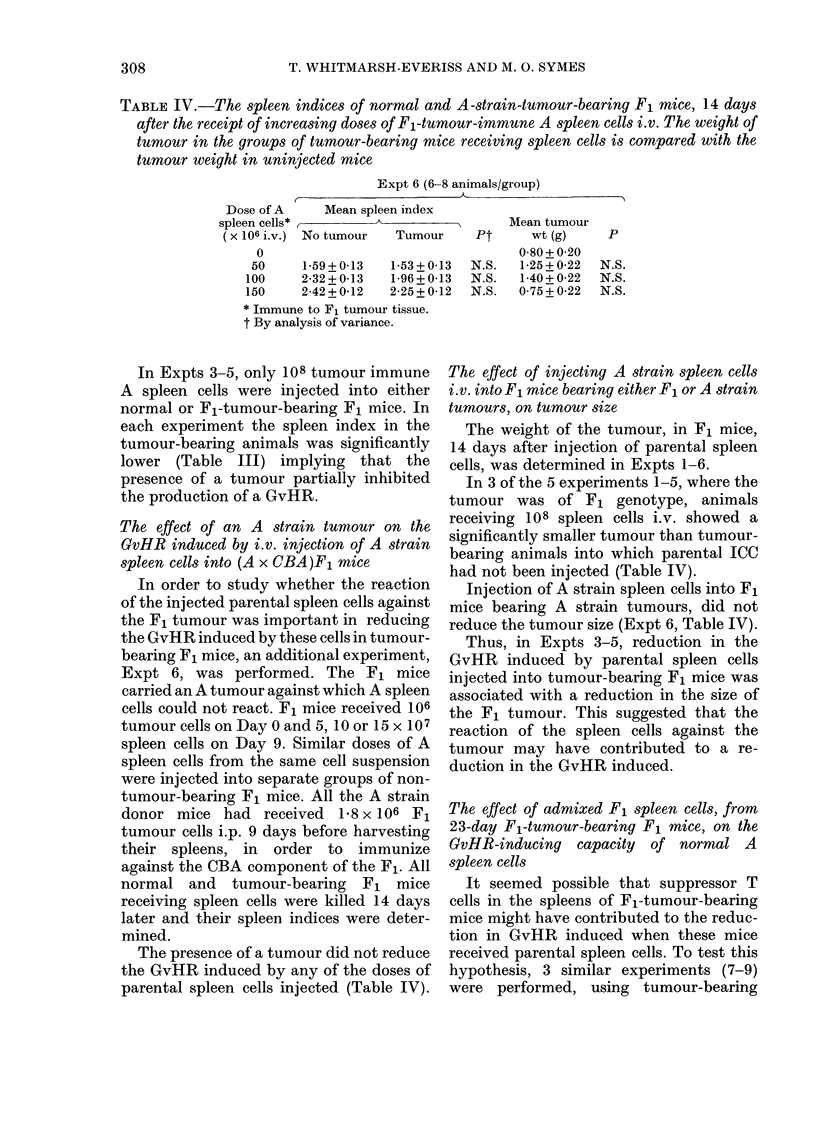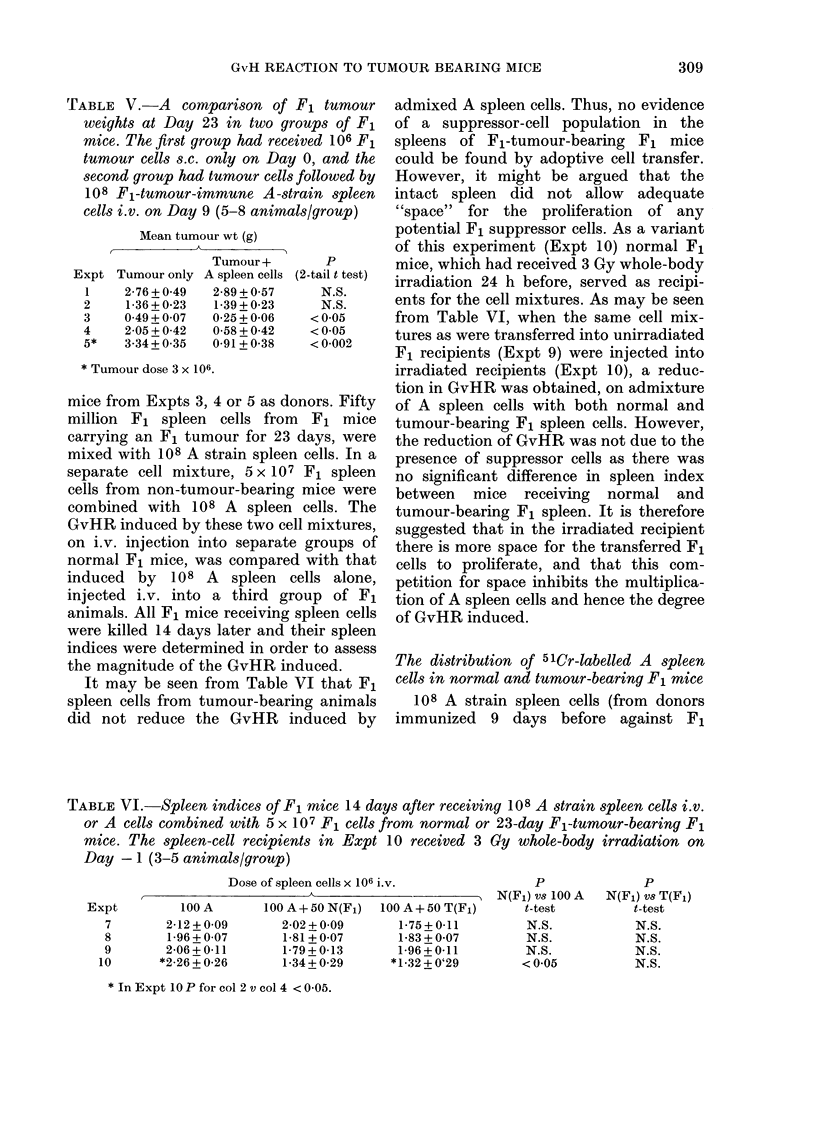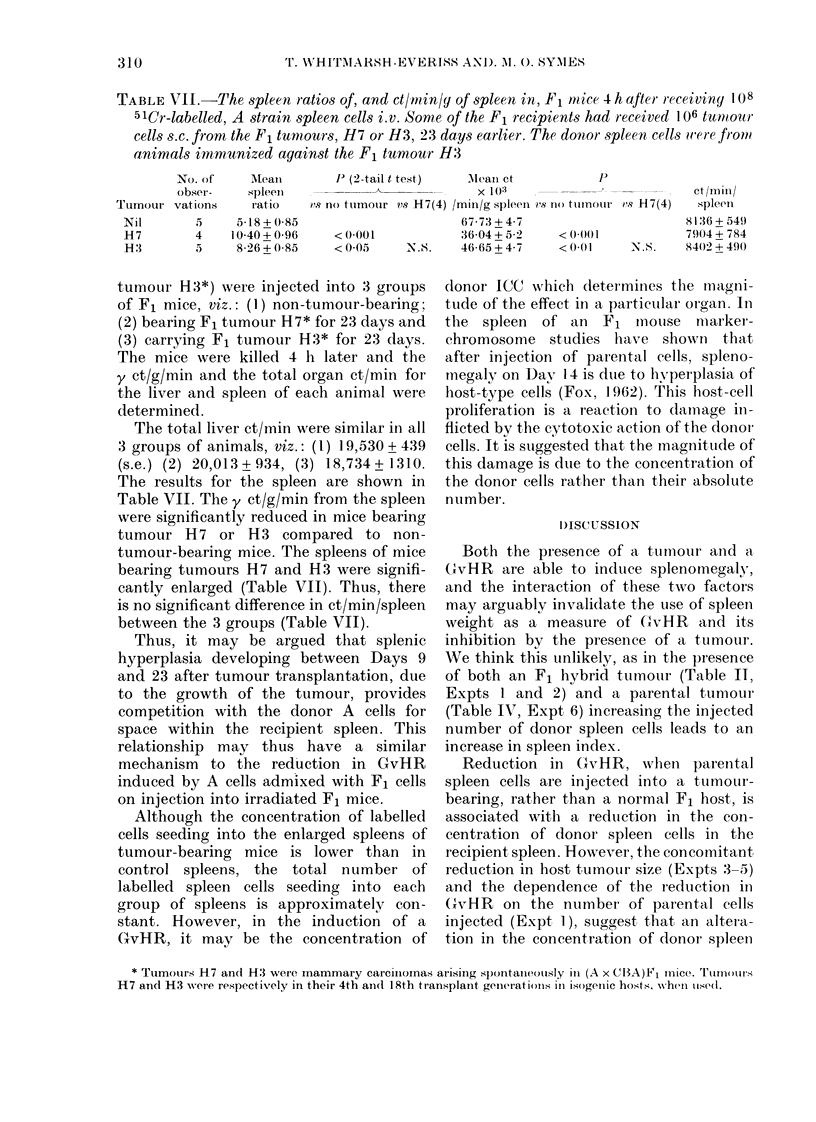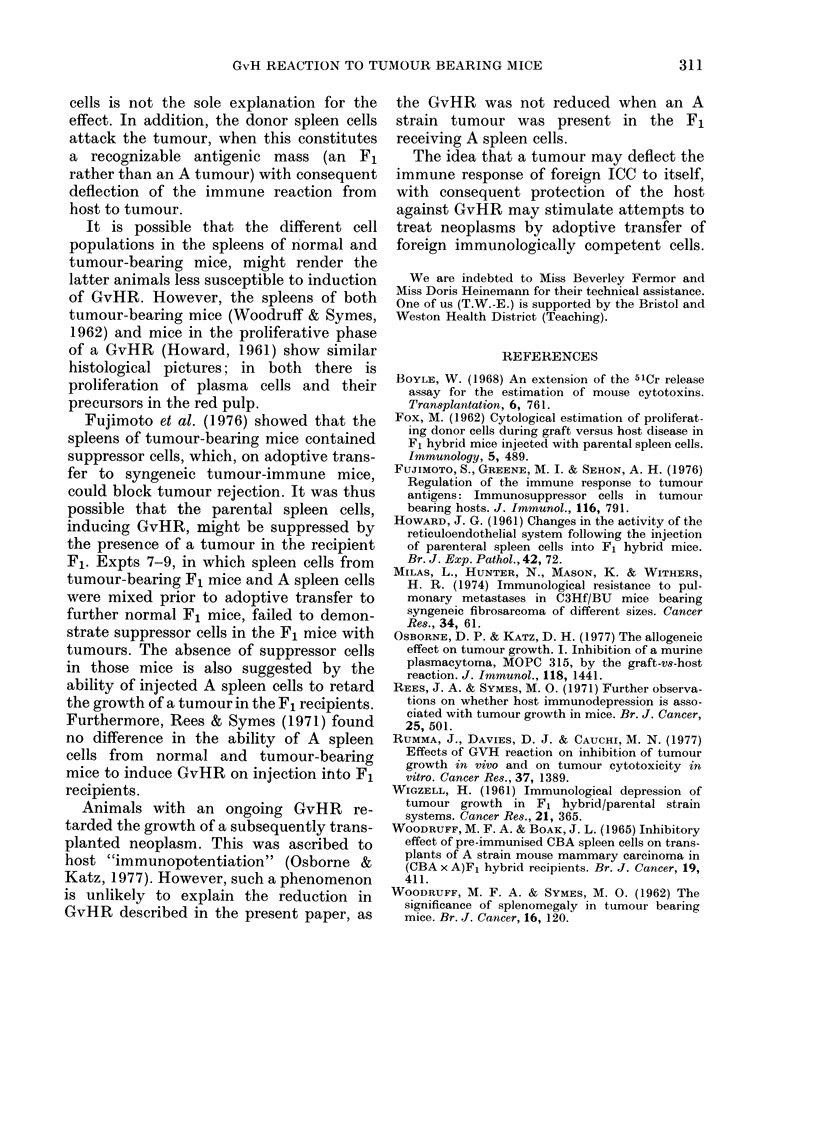# The presence of a tumour in F1 mice partially inhibits the GvH reaction following injection of parental spleen cells.

**DOI:** 10.1038/bjc.1981.48

**Published:** 1981-03

**Authors:** T. Whitmarsh-Everiss, M. O. Symes

## Abstract

(A x CBA(T6)F1 mice bearing F1 mammary carcinomas for 9 days were injected i.v. with A strain spleen cells. The A spleen cells were from either non-immune donors or mice which had received an i.p. injection of F1 tumours cells 9 days previously, and were thus immune to the CBA component of the tumour. Fourteen days after receiving parental spleen cells, the F1-tumour-bearing mice were killed and their spleen ratios and indices were determined as an index of the severity of the graft-versus-host reaction (GvHR) induced. The spleen indices were compared with those in non-tumour-bearing F1 mice, receiving aliquots of the same parental cell suspensions. At the higher doses of A spleen cells, the presence of an F1 tumour reduced the GvHR. At the same time, in 3/5 experiments, the weight of the F1 tumour in mice injected with immune A spleen cells was less than that in F1 mice receiving the same number of tumour cells but no spleen cells. A reduction in GvHR and a decrease in tumour weight was not seen when F1 mice carrying an A strain tumour were injected with A strain spleen cells immune to an F1 tumour. Adding 23-day F1 tumour-bearing F1 spleen cells to A spleen cells did not reduce the GvHR induced in further non-tumour-bearing F1 recipients by the parental cells. This was evidence against the presence of suppressor cells in the tumour-bearing F1 spleen. When 51Cr-labelled A strain spleen cells were injected into F1 mice, some of which had a tumour and therefore an enlarged spleen, there was an inverse relation between the size of the spleen and the number of parental cells therein, per g spleen 4 h after injection. It is thus suggested that the reduction in GvHR in F1-tumour-bearing F1 mice, after injection of parental spleen cells, is due first to a reduction in the concentration of donor cells in the recipient spleen (i.e. the same number of donor cells in a larger spleen) and second to pre-occupation of the donor cells in reacting to the tumour.


*Br. J. Cancer* (1981) **43**, 305

### THE PRESENCE OF A TUMOUR IN $F_1$ MICE PARTIALLY INHIBITS THE GvH REACTION FOLLOWING INJECTION OF PARENTAL SPLEEN CELLS

T. WHITMARSH-EVERISS AND M. O. SYMES

*From the Department of Surgery, The Medical School, University Walk, Bristol BS8 1TD*

Received 8 July 1980 Accepted 21 November 1980

**Summary.—(A × CBA(T6))$F_1$ mice bearing $F_1$ mammary carcinomas for 9 days were injected i.v. with A strain spleen cells. The A spleen cells were from either non-immune donors or mice which had received an i.p. injection of $F_1$ tumour cells 9 days previously, and were thus immune to the CBA component of the tumour. Fourteen days after receiving parental spleen cells, the $F_1$-tumour-bearing mice were killed and their spleen ratios and indices were determined as an index of the severity of the graft-versus-host reaction (GvHR) induced. The spleen indices were compared with those in non-tumour-bearing $F_1$ mice, receiving aliquots of the same parental cell suspensions. At the higher doses of A spleen cells, the presence of an $F_1$ tumour reduced the GvHR. At the same time, in 3/5 experiments, the weight of the $F_1$ tumour in mice injected with immune A spleen cells was less than that in $F_1$ mice receiving the same number of tumour cells but no spleen cells. A reduction in GvHR and a decrease in tumour weight was not seen when $F_1$ mice carrying an A strain tumour were injected with A strain spleen cells immune to an $F_1$ tumour. Adding 23-day $F_1$ tumour-bearing $F_1$ spleen cells to A spleen cells did not reduce the GvHR induced in further non-tumour-bearing $F_1$ recipients by the parental cells. This was evidence against the presence of suppressor cells in the tumour-bearing $F_1$ spleen.**

**When $^{51}Cr$-labelled A strain spleen cells were injected into $F_1$ mice, some of which had a tumour and therefore an enlarged spleen, there was an inverse relation between the size of the spleen and the number of parental cells therein, per g spleen 4 h after injection.**

**It is thus suggested that the reduction in GvHR in $F_1$-tumour-bearing $F_1$ mice, after injection of parental spleen cells, is due first to a reduction in the concentration of donor cells in the recipient spleen (*i.e.* the same number of donor cells in a larger spleen) and second to pre-occupation of the donor cells in reacting to the tumour.**

THERE HAVE BEEN a number of reports that injection of parental immunologically competent cells (ICC) into $F_1$ hybrid mice will inhibit the growth of tumours transplanted to these animals. There are two categories of this phenomenon, depending on whether the ICC are injected before or after tumour transplantation. Wigzell (1961) found that in 3 different $F_1$ hybrids, growth of a parental lymphoma was retarded by giving ICC from the other parent 5 days before the tumour, if the ICC were non-immune, or on the same day if the cells were tumour-immune. A similar result was obtained by Osborne & Katz (1977) using a plasmacytoma. However, in their experiment, parental ICC isogenic with the tumour also retarded tumour growth, though to a lesser extent than ICC from the other parent. Rumma *et al.* (1977) also found that ICC from the same parent as the tumour could inhibit the growth of this carcinoma in $F_1$ rats, if injected concurrently with the tumour cells or 7–14 days previously.

In contrast, Woodruff & Boak (1965) reported the inhibition of tumour growth when A strain mammary carcinomas growing in (A $\times$ CBA)$F_1$ mice were treated by 3 injections of CBA ICC (immune to the tumour) on Day 7, 14 and 21 after tumour transplantation.

There are two possible mechanisms for this effect; first, if there is a genetic difference between tumour and injected ICC, an allograft rejection reaction against the tumour, and second, in all cases, the induction of a graft-*vs*-host reaction (GvHR) with resultant host immunopotentiation (Osborne & Katz, 1977).

There is a further question raised by these studies. Can the tumour acting as an "antigenic mass" divert the action of the ICC away from the host, thus protecting the host from GvHR? We have investigated this in two tumour–host systems. First an (A $\times$ CBA)$F_1$ tumour growing in an $F_1$ host; these hybrids received parental ICC, which could thus, in theory, react against both tumour and host. Second, an A strain tumour growing in (A $\times$ CBA)$F_1$ hosts; the injected A strain ICC could now react only against the host. The resultant GvHR, measured as an increase in spleen weight, was compared between tumour-bearing and non-tumour-bearing animals in these two systems.

### MATERIALS AND METHODS

*Animals.*—Highly inbred strain A/Mi and CBA H-$T_6$(CBA) mice were maintained in this department by strict brother $\times$ sister mating of litter mates. $F_1$ animals were produced by crossing A females with CBA males.

The $F_1$ animals used as recipients of tumour transplants and parental spleen cells were aged 2–3 months.

*Tumours.*—Both A and $F_1$ mice (where the A parent is female) develop spontaneous mammary carcinomas. These tumours may be passaged serially in isogenic hosts.

Tumour-cell suspensions were prepared from these tumours by the method of Milas *et al.* (1974).

*Transplantation of tumours.*—Tumours were transplanted by injection of a cell suspension into isogenic hosts. Each recipient received 1 or $3 \times 10^6$ viable tumour cells by s.c. injection.

*Immunization of donor mice against $F_1$ tumours.*—A-strain mice aged 1–2 months each received an i.p. injection of $1{\cdot}5$–$2 \times 10^6$ tumour cells. The spleens of these mice were harvested 9 days later. In each experiment spleen cell donors and recipient mice were of the same sex.

*Preparation of spleen-cell suspensions.*—The spleen was excised and cut into small pieces which were reduced to a cell suspension by gentle grinding in a hand-operated glass-piston blender. The viable cell count was determined by dye exclusion using 0·165% w/v trypan blue as the leucocyte diluent in a haemacytometer.

*Spleen ratios and indices.*—The spleen ratio was defined as the wt of spleen (mg)/wt of mouse (g). This was evaluated on Day 14 after injection of parental cells into an $F_1$ hybrid, as Howard (1961) had shown that in the combination A->(A $\times$ CBA)$F_1$ the phagocytic index of the recipients, as a measure of GvHR, is maximal at 12–16 days. It was also demonstrated in the combination CBA or C57 BL->(CBA $\times$ C57 BL)$F_1$, that splenomegaly and an increased phagocytic index followed a similar time course. The spleen index for each animal was the quotient individual spleen ratio/mean control spleen ratio. The denominator was the mean for non-tumour-bearing or tumour-bearing mice as appropriate. In most experiments, the presence of a tumour itself induced splenomegaly (Woodruff & Symes, 1962) and this had to be considered in evaluating the increase in spleen weight due to the induction of a GvHR. Sample calculations are presented in Table I.

*Irradiation of mice.*—This was given using a 4kCi $^{137}$Cs source of $\gamma$-rays at an FSD of 60 cm. The dose rate was 0·9 rad/s.

*$^{51}$Cr labelling of spleen cells.*—Spleen-cell suspensions were prepared as described above and the contaminating erythrocytes were lysed using tris-buffered $NH_4Cl$ (Boyle, 1968).

The resulting cell suspension was then labelled with $^{51}$Cr (10$\mu$Ci of $^{51}$Cr as sodium chromate/$2 \times 10^8$ cells/ml).

### RESULTS

### *The effect of an (A $\times$ CBA)$F_1$ tumour in reducing the GvHR induced by i.v. injection of A strain spleen cells into (A $\times$ CBA)$F_1$ mice*

A total of 5 experiments were performed. In Expt 1, increasing doses of A

TABLE I.—*The method of calculating the spleen ratio and spleen index of $(A \times CBA)F_1$ normal and tumour-bearing mice,* 14 *days after they had received* $10^8$ *A spleen cells i.v. In tumour-bearing mice the weight of the tumour is subtracted from the total weight to obtain the corrected body weight, used in the calculations*

| Group | Wt of mouse + tumour (g) | Wt of tumour (g) | Wt of mouse (g) | Wt of spleen (mg) | Spleen ratio (wt of spleen/body wt) Individual | Spleen ratio mean | Spleen index = individul spleen ratio / mean control spleen ratio |
|---|---|---|---|---|---|---|---|
| (A × CBA)F1 | | | 23·18 | 88·1 | 3·80 | | |
| | | | 23·32 | 90·5 | 3·88 | | |
| | | | 24·12 | 90·5 | 3·73 | 3·86 | |
| | | | 22·52 | 86·7 | 3·84 | | |
| | | | 22·82 | 92·8 | 4·07 | | |
| | | | 24·64 | 93·9 | 3·81 | | |
| A→(A × CBA)F1 | | | 22·07 | 171·5 | 7·77 | | 2·01 |
| | | | 26·58 | 214·5 | 8·07 | | 2·09 |
| | | | etc. | | | | |
| (A × CBA)F1 with tumour | 23·34 | 1·65 | 21·69 | 181·4 | 8·36 | | |
| | 21·10 | 1·54 | 19·56 | 229·0 | 11·70 | | |
| | 23·77 | 1·26 | 22·51 | 196·3 | 8·72 | 8·34 | |
| | 23·67 | 1·32 | 22·35 | 147·6 | 6·60 | | |
| | 22·88 | 1·02 | 21·86 | 138·1 | 6·31 | | |
| A→(A × CBA)F1 with tumour | 26·34 | 1·25 | 25·09 | 243·6 | 9·71 | | 1·16 |
| | 23·09 | 1·54 | 21·55 | 241·4 | 11·20 | | 1·34 |
| | | | etc. | | | | |

TABLE II.—*The spleen indices of normal and $F_1$-tumour-bearing $(A \times CBA)F_1$ mice* 14 *days after receipt of increasing doses of non-immune or tumour-immune A spleen cells*

| Dose of A spleen cells × $10^6$ i.v. | Expt 1 Mean spleen index (± s.e.) No tumour | Expt 1 Tumour | $P$* | Expt 2 Mean spleen index No tumour | Expt 2 Tumour | $P$ |
|---|---|---|---|---|---|---|
| 50 N | 1·49 ± 0·14 | 1·14 ± 0·15 | N.S. | | | |
| 100 N | 2·15 ± 0·15 | 1·67 ± 0·15 | < 0·05 | | | |
| 50 Im | 1·86 ± 0·15 | 1·55 ± 0·17 | N.S. | 1·58 ± 0·11 | 0·87 ± 0·12 | < 0·001 |
| 100 Im | 2·22 ± 0·15 | 1·38 ± 0·17 | < 0·001 | 1·94 ± 0·11 | 1·30 ± 0·12 | < 0·001 |
| 150 Im | | | | 2·53 ± 0·11 | 1·82 ± 0·12 | < 0·001 |

N = non-immune. Im = immune to $F_1$ tumour. 5–8 animals/group.
* By analysis of variance.

spleen cells either non-immune or immune to $F_1$ tumour, were injected into $F_1$ recipients. For each dose of injected spleen cells, half the recipients had received $10^6$ $F_1$ tumour cells 9 days beforehand. All $F_1$ mice were killed 14 days after receiving A spleen cells, and their spleen indices were determined.

The presence of a tumour did not inhibit the GvHR induced by $5 \times 10^7$ non-immune or tumour-immune A spleen cells. However, when the spleen-cell dose was increased to $10^8$ cells, the spleen index of tumour-bearing mice was significantly lower in animals receiving either non-immune or immune spleen cells (Table II).

In Expt 2, the findings were similar, the presence of a tumour causing a significantly lower spleen index in $F_1$ animals receiving either 5, 10 or $15 \times 10^7$ tumour-immune parental spleen cells (Table II).

TABLE III.—*The spleen indices of normal and $F_1$-tumour-bearing $F_1$ mice* 14 *days after receipt of* $10^8$ *tumour-immune A spleen cells i.v.* (5–7 *animals/group*)

| Expt | Mean spleen index ± s.e. No tumour | Tumour | $P$ (2-tail $t$ test) |
|---|---|---|---|
| 3 | 2·12 ± 0·08 | 1·33 ± 0·07 | < 0·001 |
| 4 | 2·31 ± 0·10 | 1·60 ± 0·09 | < 0·001 |
| 5* | 1·84 ± 0·07 | 0·98 ± 0·07 | < 0·001 |

* Tumour dose $3 \times 10^6$.

TABLE IV.—*The spleen indices of normal and A-strain-tumour-bearing $F_1$ mice, 14 days after the receipt of increasing doses of $F_1$-tumour-immune A spleen cells i.v. The weight of tumour in the groups of tumour-bearing mice receiving spleen cells is compared with the tumour weight in uninjected mice*

| | Expt 6 (6–8 animals/group) | | | | |
|---|---|---|---|---|---|
| Dose of A spleen cells* ($\times 10^6$ i.v.) | Mean spleen index: No tumour | Mean spleen index: Tumour | P† | Mean tumour wt (g) | P |
| 0 | | | | $0{\cdot}80 \pm 0{\cdot}20$ | |
| 50 | $1{\cdot}59 \pm 0{\cdot}13$ | $1{\cdot}53 \pm 0{\cdot}13$ | N.S. | $1{\cdot}25 \pm 0{\cdot}22$ | N.S. |
| 100 | $2{\cdot}32 \pm 0{\cdot}13$ | $1{\cdot}96 \pm 0{\cdot}13$ | N.S. | $1{\cdot}40 \pm 0{\cdot}22$ | N.S. |
| 150 | $2{\cdot}42 \pm 0{\cdot}12$ | $2{\cdot}25 \pm 0{\cdot}12$ | N.S. | $0{\cdot}75 \pm 0{\cdot}22$ | N.S. |

* Immune to $F_1$ tumour tissue.
† By analysis of variance.

In Expts 3–5, only $10^8$ tumour immune A spleen cells were injected into either normal or $F_1$-tumour-bearing $F_1$ mice. In each experiment the spleen index in the tumour-bearing animals was significantly lower (Table III) implying that the presence of a tumour partially inhibited the production of a GvHR.

*The effect of an A strain tumour on the GvHR induced by i.v. injection of A strain spleen cells into $(A \times CBA)F_1$ mice*

In order to study whether the reaction of the injected parental spleen cells against the $F_1$ tumour was important in reducing the GvHR induced by these cells in tumour-bearing $F_1$ mice, an additional experiment, Expt 6, was performed. The $F_1$ mice carried an A tumour against which A spleen cells could not react. $F_1$ mice received $10^6$ tumour cells on Day 0 and 5, 10 or $15 \times 10^7$ spleen cells on Day 9. Similar doses of A spleen cells from the same cell suspension were injected into separate groups of non-tumour-bearing $F_1$ mice. All the A strain donor mice had received $1{\cdot}8 \times 10^6$ $F_1$ tumour cells i.p. 9 days before harvesting their spleens, in order to immunize against the CBA component of the $F_1$. All normal and tumour-bearing $F_1$ mice receiving spleen cells were killed 14 days later and their spleen indices were determined.

The presence of a tumour did not reduce the GvHR induced by any of the doses of parental spleen cells injected (Table IV).

*The effect of injecting A strain spleen cells i.v. into $F_1$ mice bearing either $F_1$ or A strain tumours, on tumour size*

The weight of the tumour, in $F_1$ mice, 14 days after injection of parental spleen cells, was determined in Expts 1–6.

In 3 of the 5 experiments 1–5, where the tumour was of $F_1$ genotype, animals receiving $10^8$ spleen cells i.v. showed a significantly smaller tumour than tumour-bearing animals into which parental ICC had not been injected (Table IV).

Injection of A strain spleen cells into $F_1$ mice bearing A strain tumours, did not reduce the tumour size (Expt 6, Table IV).

Thus, in Expts 3–5, reduction in the GvHR induced by parental spleen cells injected into tumour-bearing $F_1$ mice was associated with a reduction in the size of the $F_1$ tumour. This suggested that the reaction of the spleen cells against the tumour may have contributed to a reduction in the GvHR induced.

*The effect of admixed $F_1$ spleen cells, from 23-day $F_1$-tumour-bearing $F_1$ mice, on the GvHR-inducing capacity of normal A spleen cells*

It seemed possible that suppressor T cells in the spleens of $F_1$-tumour-bearing mice might have contributed to the reduction in GvHR induced when these mice received parental spleen cells. To test this hypothesis, 3 similar experiments (7–9) were performed, using tumour-bearing

TABLE V.—*A comparison of $F_1$ tumour weights at Day 23 in two groups of $F_1$ mice. The first group had received $10^6$ $F_1$ tumour cells s.c. only on Day 0, and the second group had tumour cells followed by $10^8$ $F_1$-tumour-immune A-strain spleen cells i.v. on Day 9 (5–8 animals/group)*

| | Mean tumour wt (g) | | |
|---|---|---|---|
| Expt | Tumour only | Tumour + A spleen cells | P (2-tail t test) |
| 1 | 2·76 ± 0·49 | 2·89 ± 0·57 | N.S. |
| 2 | 1·36 ± 0·23 | 1·39 ± 0·23 | N.S. |
| 3 | 0·49 ± 0·07 | 0·25 ± 0·06 | < 0·05 |
| 4 | 2·05 ± 0·42 | 0·58 ± 0·42 | < 0·05 |
| 5* | 3·34 ± 0·35 | 0·91 ± 0·38 | < 0·002 |

* Tumour dose $3 \times 10^6$.

mice from Expts 3, 4 or 5 as donors. Fifty million $F_1$ spleen cells from $F_1$ mice carrying an $F_1$ tumour for 23 days, were mixed with $10^8$ A strain spleen cells. In a separate cell mixture, $5 \times 10^7$ $F_1$ spleen cells from non-tumour-bearing mice were combined with $10^8$ A spleen cells. The GvHR induced by these two cell mixtures, on i.v. injection into separate groups of normal $F_1$ mice, was compared with that induced by $10^8$ A spleen cells alone, injected i.v. into a third group of $F_1$ animals. All $F_1$ mice receiving spleen cells were killed 14 days later and their spleen indices were determined in order to assess the magnitude of the GvHR induced.

It may be seen from Table VI that $F_1$ spleen cells from tumour-bearing animals did not reduce the GvHR induced by admixed A spleen cells. Thus, no evidence of a suppressor-cell population in the spleens of $F_1$-tumour-bearing $F_1$ mice could be found by adoptive cell transfer. However, it might be argued that the intact spleen did not allow adequate "space" for the proliferation of any potential $F_1$ suppressor cells. As a variant of this experiment (Expt 10) normal $F_1$ mice, which had received 3 Gy whole-body irradiation 24 h before, served as recipients for the cell mixtures. As may be seen from Table VI, when the same cell mixtures as were transferred into unirradiated $F_1$ recipients (Expt 9) were injected into irradiated recipients (Expt 10), a reduction in GvHR was obtained, on admixture of A spleen cells with both normal and tumour-bearing $F_1$ spleen cells. However, the reduction of GvHR was not due to the presence of suppressor cells as there was no significant difference in spleen index between mice receiving normal and tumour-bearing $F_1$ spleen. It is therefore suggested that in the irradiated recipient there is more space for the transferred $F_1$ cells to proliferate, and that this competition for space inhibits the multiplication of A spleen cells and hence the degree of GvHR induced.

*The distribution of $^{51}$Cr-labelled A spleen cells in normal and tumour-bearing $F_1$ mice*

$10^8$ A strain spleen cells (from donors immunized 9 days before against $F_1$

TABLE VI.—*Spleen indices of $F_1$ mice 14 days after receiving $10^8$ A strain spleen cells i.v. or A cells combined with $5 \times 10^7$ $F_1$ cells from normal or 23-day $F_1$-tumour-bearing $F_1$ mice. The spleen-cell recipients in Expt 10 received 3 Gy whole-body irradiation on Day −1 (3–5 animals/group)*

| | Dose of spleen cells × $10^6$ i.v. | | | P | P |
|---|---|---|---|---|---|
| Expt | 100 A | 100 A + 50 N($F_1$) | 100 A + 50 T($F_1$) | N($F_1$) *vs* 100 A *t*-test | N($F_1$) *vs* T($F_1$) *t*-test |
| 7 | 2·12 ± 0·09 | 2·02 ± 0·09 | 1·75 ± 0·11 | N.S. | N.S. |
| 8 | 1·96 ± 0·07 | 1·81 ± 0·07 | 1·83 ± 0·07 | N.S. | N.S. |
| 9 | 2·06 ± 0·11 | 1·79 ± 0·13 | 1·96 ± 0·11 | N.S. | N.S. |
| 10 | *2·26 ± 0·26 | 1·34 ± 0·29 | *1·32 ± 0‘29 | < 0·05 | N.S. |

* In Expt 10 *P* for col 2 *v* col 4 < 0·05.

TABLE VII.—*The spleen ratios of, and ct/min/g of spleen in, $F_1$ mice 4 h after receiving* $10^8$ $^{51}$*Cr-labelled, A strain spleen cells i.v. Some of the $F_1$ recipients had received* $10^6$ *tumour cells s.c. from the $F_1$ tumours, H7 or H3, 23 days earlier. The donor spleen cells were from animals immunized against the $F_1$ tumour H3*

| Tumour | No. of observations | Mean spleen ratio | P (2-tail t test) vs no tumour | P (2-tail t test) vs H7(4) | Mean ct × $10^3$ /min/g spleen | P vs no tumour | P vs H7(4) | ct/min/spleen |
|---|---|---|---|---|---|---|---|---|
| Nil | 5 | 5·18 ± 0·85 | | | 67·73 ± 4·7 | | | 8136 ± 549 |
| H7 | 4 | 10·40 ± 0·96 | < 0·001 | | 36·04 ± 5·2 | < 0·001 | | 7904 ± 784 |
| H3 | 5 | 8·26 ± 0·85 | < 0·05 | N.S. | 46·65 ± 4·7 | < 0·01 | N.S. | 8402 ± 490 |

tumour H3*) were injected into 3 groups of $F_1$ mice, *viz.*: (1) non-tumour-bearing; (2) bearing $F_1$ tumour H7* for 23 days and (3) carrying $F_1$ tumour H3* for 23 days. The mice were killed 4 h later and the $\gamma$ ct/g/min and the total organ ct/min for the liver and spleen of each animal were determined.

The total liver ct/min were similar in all 3 groups of animals, *viz.*: (1) 19,530 ± 439 (s.e.) (2) 20,013 ± 934, (3) 18,734 ± 1310. The results for the spleen are shown in Table VII. The $\gamma$ ct/g/min from the spleen were significantly reduced in mice bearing tumour H7 or H3 compared to non-tumour-bearing mice. The spleens of mice bearing tumours H7 and H3 were significantly enlarged (Table VII). Thus, there is no significant difference in ct/min/spleen between the 3 groups (Table VII).

Thus, it may be argued that splenic hyperplasia developing between Days 9 and 23 after tumour transplantation, due to the growth of the tumour, provides competition with the donor A cells for space within the recipient spleen. This relationship may thus have a similar mechanism to the reduction in GvHR induced by A cells admixed with $F_1$ cells on injection into irradiated $F_1$ mice.

Although the concentration of labelled cells seeding into the enlarged spleens of tumour-bearing mice is lower than in control spleens, the total number of labelled spleen cells seeding into each group of spleens is approximately constant. However, in the induction of a GvHR, it may be the concentration of donor ICC which determines the magnitude of the effect in a particular organ. In the spleen of an $F_1$ mouse marker-chromosome studies have shown that after injection of parental cells, splenomegaly on Day 14 is due to hyperplasia of host-type cells (Fox, 1962). This host-cell proliferation is a reaction to damage inflicted by the cytotoxic action of the donor cells. It is suggested that the magnitude of this damage is due to the concentration of the donor cells rather than their absolute number.

### DISCUSSION

Both the presence of a tumour and a GvHR are able to induce splenomegaly, and the interaction of these two factors may arguably invalidate the use of spleen weight as a measure of GvHR and its inhibition by the presence of a tumour. We think this unlikely, as in the presence of both an $F_1$ hybrid tumour (Table II, Expts 1 and 2) and a parental tumour (Table IV, Expt 6) increasing the injected number of donor spleen cells leads to an increase in spleen index.

Reduction in GvHR, when parental spleen cells are injected into a tumour-bearing, rather than a normal $F_1$ host, is associated with a reduction in the concentration of donor spleen cells in the recipient spleen. However, the concomitant reduction in host tumour size (Expts 3–5) and the dependence of the reduction in GvHR on the number of parental cells injected (Expt 1), suggest that an alteration in the concentration of donor spleen

* Tumours H7 and H3 were mammary carcinomas arising spontaneously in (A × CBA)$F_1$ mice. Tumours H7 and H3 were respectively in their 4th and 18th transplant generations in isogenic hosts, when used.

cells is not the sole explanation for the effect. In addition, the donor spleen cells attack the tumour, when this constitutes a recognizable antigenic mass (an $F_1$ rather than an A tumour) with consequent deflection of the immune reaction from host to tumour.

It is possible that the different cell populations in the spleens of normal and tumour-bearing mice, might render the latter animals less susceptible to induction of GvHR. However, the spleens of both tumour-bearing mice (Woodruff & Symes, 1962) and mice in the proliferative phase of a GvHR (Howard, 1961) show similar histological pictures; in both there is proliferation of plasma cells and their precursors in the red pulp.

Fujimoto *et al.* (1976) showed that the spleens of tumour-bearing mice contained suppressor cells, which, on adoptive transfer to syngeneic tumour-immune mice, could block tumour rejection. It was thus possible that the parental spleen cells, inducing GvHR, might be suppressed by the presence of a tumour in the recipient $F_1$. Expts 7–9, in which spleen cells from tumour-bearing $F_1$ mice and A spleen cells were mixed prior to adoptive transfer to further normal $F_1$ mice, failed to demonstrate suppressor cells in the $F_1$ mice with tumours. The absence of suppressor cells in those mice is also suggested by the ability of injected A spleen cells to retard the growth of a tumour in the $F_1$ recipients. Furthermore, Rees & Symes (1971) found no difference in the ability of A spleen cells from normal and tumour-bearing mice to induce GvHR on injection into $F_1$ recipients.

Animals with an ongoing GvHR retarded the growth of a subsequently transplanted neoplasm. This was ascribed to host "immunopotentiation" (Osborne & Katz, 1977). However, such a phenomenon is unlikely to explain the reduction in GvHR described in the present paper, as the GvHR was not reduced when an A strain tumour was present in the $F_1$ receiving A spleen cells.

The idea that a tumour may deflect the immune response of foreign ICC to itself, with consequent protection of the host against GvHR may stimulate attempts to treat neoplasms by adoptive transfer of foreign immunologically competent cells.

We are indebted to Miss Beverley Fermor and Miss Doris Heinemann for their technical assistance. One of us (T.W.-E.) is supported by the Bristol and Weston Health District (Teaching).

### REFERENCES

BOYLE, W. (1968) An extension of the $^{51}$Cr release assay for the estimation of mouse cytotoxins. *Transplantation*, **6**, 761.

FOX, M. (1962) Cytological estimation of proliferating donor cells during graft versus host disease in $F_1$ hybrid mice injected with parental spleen cells. *Immunology*, **5**, 489.

FUJIMOTO, S., GREENE, M. I. & SEHON, A. H. (1976) Regulation of the immune response to tumour antigens: Immunosuppressor cells in tumour bearing hosts. *J. Immunol.*, **116**, 791.

HOWARD, J. G. (1961) Changes in the activity of the reticuloendothelial system following the injection of parenteral spleen cells into $F_1$ hybrid mice. *Br. J. Exp. Pathol.*, **42**, 72.

MILAS, L., HUNTER, N., MASON, K. & WITHERS, H. R. (1974) Immunological resistance to pulmonary metastases in C3Hf/BU mice bearing syngeneic fibrosarcoma of different sizes. *Cancer Res.*, **34**, 61.

OSBORNE, D. P. & KATZ, D. H. (1977) The allogeneic effect on tumour growth. I. Inhibition of a murine plasmacytoma, MOPC 315, by the graft-*vs*-host reaction. *J. Immunol.*, **118**, 1441.

REES, J. A. & SYMES, M. O. (1971) Further observations on whether host immunodepression is associated with tumour growth in mice. *Br. J. Cancer*, **25**, 501.

RUMMA, J., DAVIES, D. J. & CAUCHI, M. N. (1977) Effects of GVH reaction on inhibition of tumour growth *in vivo* and on tumour cytotoxicity *in vitro*. *Cancer Res.*, **37**, 1389.

WIGZELL, H. (1961) Immunological depression of tumour growth in $F_1$ hybrid/parental strain systems. *Cancer Res.*, **21**, 365.

WOODRUFF, M. F. A. & BOAK, J. L. (1965) Inhibitory effect of pre-immunised CBA spleen cells on transplants of A strain mouse mammary carcinoma in (CBA × A)$F_1$ hybrid recipients. *Br. J. Cancer*, **19**, 411.

WOODRUFF, M. F. A. & SYMES, M. O. (1962) The significance of splenomegaly in tumour bearing mice. *Br. J. Cancer*, **16**, 120.